# Mannan-Binding Lectin Levels and Activity Are Not Altered in Atopic Dermatitis Patients with a History of Eczema Herpeticum

**DOI:** 10.1155/2011/769890

**Published:** 2011-11-10

**Authors:** Kemp W. Bundy, Laura Y. McGirt, Lora G. Bankova, Andreas Wollenberg, Lisa A. Beck, Anna De Benedetto

**Affiliations:** ^1^Division of Allergy, Immunology, Rheumatology, University of Rochester Medical Center, Rochester, NY 14642, USA; ^2^Department of Dermatology, Johns Hopkins University, Baltimore, MD 21205, USA; ^3^Department of Dermatology and Allergy, Ludwig Maximilian University of Munich, 80539 Munich, Germany; ^4^Department of Dermatology, University of Rochester Medical Center, 601 Elmwood Avenue, P.O. Box 697, Rochester, NY 14642, USA

## Abstract

*Background*. Eczema herpeticum (EH) is a potentially serious, systemic complication in subjects with atopic dermatitis (AD) caused by herpes simplex virus (HSV). The innate immune dysregulation that predisposes these subjects to cutaneous viral infections is not well understood. We tested the hypothesis that defects in mannan-binding lectin (MBL) may be associated with an increased risk of EH. *Methods*. We evaluated serum MBL levels and functional activity in 13 AD subjects with a history of EH (EH+) and 21 AD subjects with no history of EH (EH−). MBL levels were detected by enzyme immunoassay. MBL pathway functional activity was evaluated by determining MBL C4b deposition capacity. *Results*. We found no statistical difference in MBL serum levels or function between EH+ and EH− groups. *Conclusion*. Considering the limitations of this study (e.g., small samples size) our findings suggest that MBL defects do not play a role in EH.

## 1. Introduction

Atopic dermatitis (AD) is the most common chronic, inflammatory skin disease. Although rare, one of the most severe complications of AD is a widely disseminated cutaneous infection with HSV, called eczema herpeticum (EH) [1]. Episodes of EH can be complicated by keratoconjunctivitis and viremia, leading to multiple organ involvement including meningitis and encephalitis. This makes EH one of the few true emergencies in dermatology [2].

Recently, it has been shown that AD subjects with a history of EH have a more severe phenotype as compared to AD subjects without a history of this viral complication. ADEH+ patients have greater disease severity index score, greater allergen sensitization, and higher total IgE levels and peripheral eosinophilia [3]. 

The major hypothesis put forth to explain AD subjects' susceptibility to HSV and other viral infections is that they have one or more defects in the innate immune system. Thus far, the specific mechanism responsible for this condition has not been determined. Numerous publications have documented that the epidermis of AD subjects has reduced expression of antimicrobial proteins. Howell et al. [4] found that the LL-37-deficient (Clnp−/−) mice had greater HSV replication on the skin surface than wild-type mice, suggesting that the lack of this antimicrobial peptide may provide an explanation for AD patients' predisposition to EH. Another hypothesis contends that the impaired recruitment and function of plasmacytoid dendritic cells (PDC), which produce antiviral type 1 interferons, would predispose AD subjects to viral skin infections [1]. Recently, we have also highlighted that the greater viral susceptibility may be related to a defect in epidermal tight junctions which is observed in AD subjects [5].

There is preliminary epidemiologic evidence to suggest that Mannan-Binding Lectin (MBL) is important for clearance of several common viruses (hepatitis B, HIV, and influenza A) [6]. In this study we tested the hypothesis that defects in MBL level or activity may be associated with an increased risk for increased risk for EH.

MBL, a soluble molecule from the collectin family of pathogen-related receptors, is a C-type lectin of hepatic origin that binds and circulates in a complex with MBL-associated serine proteases (MASPs). MBL-MASP2 initiates complement activation and microbial opsonization resulting in phagocytosis of foreign pathogens. 

The expression of functional MBL protein is largely genetically determined [7]. Serum MBL deficiency has been observed in 10 to 15% of Caucasians with significantly higher percentages reported in subjects of African or South American Indian descent [6]. Such deficiencies and their variant alleles have been associated with increased susceptibility to bacterial infections in neutropenic subjects, poorer prognosis in cystic fibrosis patients, or more severe meningococcal disease [6]. 

Interestingly, Seppänen et al. [8] demonstrated an *MBL2 *(MBL gene) structural variant genotype (A/O or O/O) that was more common among the patients with recurrent HSV-2 infection compared with healthy controls or controls positive for HSV-2 antibodies. A recent report highlights a clear association between extremely low MBL levels and the BB MBL haplotype in several members of a Turkish family who also suffered from recurrent staphylococcal infections (skin, ear, and airway) and a pruritic, eczematous dermatitis [9]. Two genetic studies have reported conflicting results regarding MBL polymorphisms and their associations with AD. Brandrup et al. [9] did not find an association between the MBL B allele and AD susceptibility in a Japanese population. In contrast, a study of Brazilian AD subjects demonstrated that the three exon 1 variants (B, C, and D) were observed more frequently than in a healthy control population [10].

No studies have evaluated MBL levels or genotypes in AD EH+ subjects.

## 2. Materials and Methods

### 2.1. Study Population

This study received approval from the Johns Hopkins Medical Institution IRB as well as by the ethics committee of the Department of Dermatology and Allergy, Ludwig Maximilian University, Munich, Germany. Written informed consent was obtained. Serum was collected from adult subjects whose diagnosis of AD was made by a dermatologist who specializes in the care of AD (LAB and AW; *n* = 34). AD subjects were categorized as EH+ if the subject had an episode of EH that was observed by one of the two dermatologists and for which the presence of HSV was confirmed by one of the following means (culture, Tzanck smear, PCR, or immunofluorescence assay). Subjects in the control AD group were selected if they had no history of EH as assessed by a careful history (EH−) and had never been prescribed systemic antiviral therapy.

### 2.2. MBL Serum Levels and Functional Assay

Serum MBL levels were detected by enzyme immunoassay using monoclonal antibody against the oligomeric MBL carbohydrate-binding domain. MBL pathway functional activity was evaluated indirectly by determining MBL C4b deposition capacity with an anti-human C4 monoclonal antibody. Both of these studies were performed at IBT reference laboratory (Lenexa, Kansas).

### 2.3. Statistical Analysis

Differences between groups were evaluated using a 2-tailed, unpaired *t*-test. A *P* value of ≤0.05 was considered statistically significant. Data were expressed as mean ± SEM.

## 3. Results and Discussion

**Figure 1:**
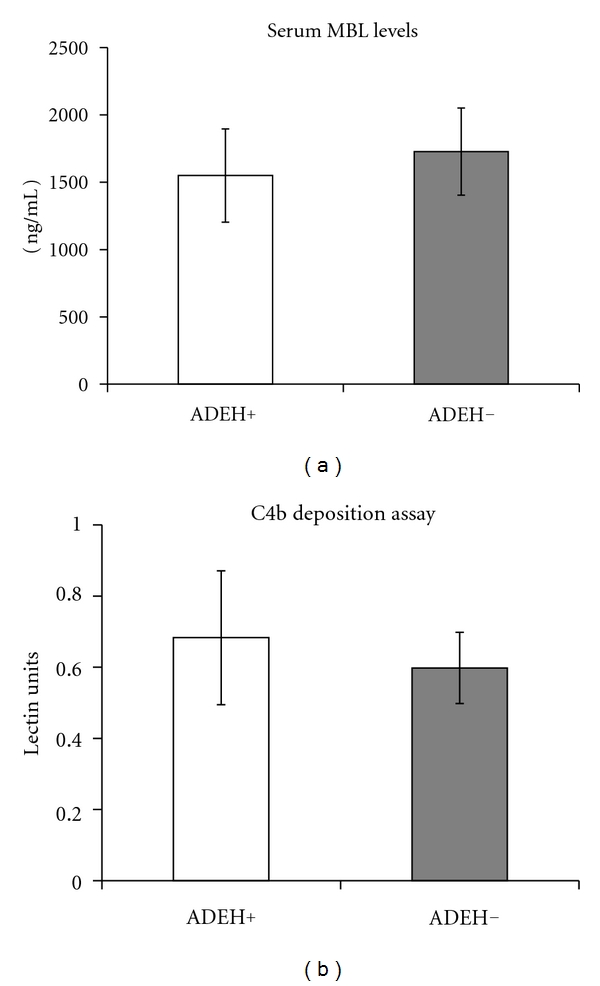
Mannan-binding lectin levels and activity in atopic dermatitis patients with and without a history of eczema herpeticum are not different. (a) Serum levels of MBL (ng/mL; ref. range >200 ng/mL) and (b) MBL functional activity (lectin units; ref. range >0.2 lectin units) in ADEH+ (*n* = 13) or ADEH− (*n* = 21) patients. Red line represents lower limit of the normal reference range.

The EH− cohort (*n* = 21) had a mean age of 30 ± 4 years and 16 of 21 subjects (76%) were female, whereas the EH+ cohort (*n* = 13) had a mean age of 36 ± 5 years and 4 of 13 subjects (31%) were female. The mean serum MBL level (ref. range >200 ng/mL) for EH− patients was 1727 ± 323 ng/mL and for EH+ patients was 1549 ± 346 ng/mL (*P* = 0.703) (Figure 1(a)). MBL functional activity as measured by C4b deposition assay (ref. range >0.2 lectin units) for EH− patients was 0.6 ± 0.1 lectin units and for EH+ patients was 0.7 ± 0.1 lectin units (*P* = 0.685) (Figure 1(b)). There was no statistically significant difference in MBL levels or functional activity between the two AD groups (EH− and EH+).

 Our study demonstrates that AD subjects with a history of EH had similar levels and functional activity of serum MBL as AD subjects with no history of EH. This suggests that MBL deficiency or defects in this pathway are not likely to be a susceptibility factor for the development of EH. Evaluation of this in a larger cohort of AD patients, as well as in ADEH+ subjects during an acute EH event, would be needed to solidify this conclusion.

## 4. Conclusions

Clinical studies aiming to clarify the pathogenesis of EH in AD are limited by the low prevalence of the disease and often by the very young age of patients involved. Additionally, a clinical test that can carefully characterize the subphenotype after the acute event is not available, thus making it difficult to study very well-characterized populations. Considering these limitations, we believe that any data, even negative, should be made available to other investigators in the field. Further work is needed to determine the precise defect(s) that predispose AD patients to this potentially life-threatening viral skin infection.
